# 1 A Preliminary Analysis of Transaminitis Observed in Oxandrolone Versus Testosterone Therapy in Major Burn Injury

**DOI:** 10.1093/jbcr/iraf019.001

**Published:** 2025-04-01

**Authors:** Alexandra DeWitt, Athena Hoppe, Anastasiya Ivanko, Victoria Miles

**Affiliations:** University Medical Center New Orleans; Louisiana State University; Burn Center at University Medical Center; University Medical Center New Orleans

## Abstract

**Introduction:**

After major burn injury, patients experience a hypermetabolic response leading to catabolic effects. Anabolic steroids have been investigated to combat these effects. Oxandrolone, the primary anabolic steroid used to combat burn hypermetabolism, was removed from the U.S. market in June 2023, and testosterone has been implemented as an alternative. A known side effect of anabolic steroid use is transaminitis. This study aims to compare the incidence of transaminitis between oxandrolone and testosterone in patients with major burn injury.

**Methods:**

A single-center, retrospective cohort was conducted to evaluate adult patients with at least 20% TBSA burn injury who received either testosterone or oxandrolone. The primary outcome evaluated was incidence of transaminitis. Secondary outcomes included the need for dose reduction or discontinuation of the steroid, length of stay, and mortality. Preliminary data was analyzed for significance.

**Results:**

Seventy patients received either oxandrolone (n=52) or testosterone (n=18). Demographics were similar. The incidence of transaminitis was not statistically significant between oxandrolone and testosterone, 38% vs 28% (p = 0.596). The rate of dose decrease between the two groups was not significant, 17% vs 0% (p = 1.138). There was a statistically significant difference in early discontinuation of the drug between the groups, 33% oxandrolone vs 0% testosterone (p = 0.014). The median length of stay was 28 and 36 days, respectively, with a mortality rate of 21% and 6% in each group.

**Conclusions:**

Preliminary data from this study demonstrate a trend to higher incidence of oxandrolone transaminitis in comparison to testosterone, without statistical significance.

**Applicability of Research to Practice:**

This study shows that the incidence of clinically significant transaminitis with a liver function test increase more than three times the upper limit of normal may be less with testosterone over historical oxandrolone.

**Funding for the Study:**

N/A

